# CircSSNN: circRNA-binding site prediction via sequence self-attention neural networks with pre-normalization

**DOI:** 10.1186/s12859-023-05352-7

**Published:** 2023-05-30

**Authors:** Chao Cao, Shuhong Yang, Mengli Li, Chungui Li

**Affiliations:** 1 School of Computer Science and Technology, Guangxi University of Science and Technology, Liuzhou, China; 2 Key Laboratory of Guangxi Universities on Intelligent Computing and Distributed Information Processing, Guangxi University of Science and Technology, Liuzhou, China; 3 School of Technology, Guilin University, Guilin, China

**Keywords:** Circular RNAs, RNA-binding proteins, CircRNA-binding site prediction, Self-attention neural networks, Pre-normalization

## Abstract

**Background:**

Circular RNAs (circRNAs) play a significant role in some diseases by acting as transcription templates. Therefore, analyzing the interaction mechanism between circRNA and RNA-binding proteins (RBPs) has far-reaching implications for the prevention and treatment of diseases. Existing models for circRNA-RBP identification usually adopt convolution neural network (CNN), recurrent neural network (RNN), or their variants as feature extractors. Most of them have drawbacks such as poor parallelism, insufficient stability, and inability to capture long-term dependencies.

**Methods:**

In this paper, we propose a new method completely using the self-attention mechanism to capture deep semantic features of RNA sequences. On this basis, we construct a CircSSNN model for the cirRNA-RBP identification. The proposed model constructs a feature scheme by fusing circRNA sequence representations with statistical distributions, static local contexts, and dynamic global contexts. With a stable and efficient network architecture, the distance between any two positions in a sequence is reduced to a constant, so CircSSNN can quickly capture the long-term dependencies and extract the deep semantic features.

**Results:**

Experiments on 37 circRNA datasets show that the proposed model has overall advantages in stability, parallelism, and prediction performance. Keeping the network structure and hyperparameters unchanged, we directly apply the CircSSNN to linRNA datasets. The favorable results show that CircSSNN can be transformed simply and efficiently without task-oriented tuning.

**Conclusions:**

In conclusion, CircSSNN can serve as an appealing circRNA-RBP identification tool with good identification performance, excellent scalability, and wide application scope without the need for task-oriented fine-tuning of parameters, which is expected to reduce the professional threshold required for hyperparameter tuning in bioinformatics analysis.

## Introduction

Circular RNA (or circRNA) is a single-stranded RNA with a closed-loop structure [1, 2]. It is resistant to exonuclease-mediated degradation, and is more stable than most linear RNA. Recent studies have shown that circRNA molecules are rich in microRNA (miRNA) binding sites, which act as miRNA sponge (miRNA sponge) in cells [3–5], thus relieving the repressive effect of miRNA on its target genes and increasing the expression level of target genes. This mechanism of action is known as a competitive endogenous RNA (ceRNA) mechanism. By interacting with disease-associated miRNAs, circRNA plays a significant role in disease [6–8]. It has been shown that circRNA is conducive to the suppression of cancer by binding to some RBPs [9]. Therefore, an in-depth analysis of the interaction between circRNAs and RBPs to understand the development of tumor biology has a remarkable significance.

Benefiting from the high-throughput sequencing of RNA isolated by crosslinking immunoprecipitation (HITS-CLIP, also known as CLIP-Seq) sequencing technology, researchers have found there are several RBP binding sites in circRNA in eukaryotes [10, 11]. Therefore, many bioinformatic methods have been proposed to predict circRNA-RBP interactions. For example, inspired by the extraction of image features, Wang et al. proposed a circRNA-RBP classification model based on CNN, which uses the RBP binding sites on CS-circRNAs to predict its relevance to cancer [12]. Based on the capsule network, the CircRB [13] model also utilized convolutional operations to extract the features of circRNAs, and leveraged the dynamic routing algorithm to classify the binding sites. To introduce temporal information in circRNA-protein binding sites, Ju et al. first used CNN to extract features, then combined LSTM with conditional random fields and proposed a sequence-tagged deep learning model to identify circRNA-protein binding sites [14]. Similarly, Zhang et al. combined CNN and BiLSTM into a hybrid neural network in the CRIP model [15]. They also use CNN to extract features and use BiLSTM to capture the temporal information and obtain long-term association information. Unlike the methods mentioned above, CRIP used a codon-based scheme to encode RNA sequences [15]. Also based on a hybrid deep network composed of CNN and BiLSTM networks, Jia et al. applied XGBoost with incremental feature selection to conduct feature encoding and proposed PASSION [16] algorithm for circRNA-protein binding site prediction. Drawing on the ideas of NLP, Yang et al. proposed a KNFP (K-tuple Nucleotide Frequency Pattern) encoding scheme to describe local information, and applied word2vec to obtain global statistical information. The network architecture in Yang’s model is a hybrid model consisting of a multi-scale residual CNN, a BiGRU network and the attention Mechanism [17]. On this basis, Circ2CBA [18] uses a one-hot method to encode circRNA sequences and replaces the BiGRU network with BiLSTM. DeCban [19] combines CNNs with Attention Networks directly for feature extraction. Li et al. and Niu et al. introduced multi-view subspace learning and ensemble neural network into Yang’s model, and proposed two models named as DMSK [20] and CRBPDL [21], respectively. The models mentioned above have made impressive improvements in the performance of circRNA-RBP prediction, but there are still limitations in the description of global relations. This is because that these methods fail to make full use of the contextual information of circRNA sequences.

To overcome this issue, inspired by the newly proposed BERT(Bidirectional Encoder Representations from Transformers) model, Yang et.al first pre-trained a DNABERT model [22], then fine-tuned the DNABERT to capture the semantic and syntactic information of the initial RNA sequence, and finally used the deep temporal convolutional network(DTCN) to predict the circRNA-protein binding sites [23]. Though the existing models have made many attempts, from single-view to multi-view, to enrich the diversity of features, they mainly resort to CNN and RNN or a hybrid of them to extract the deep features of circRNA, there is still large room for improvements regarding the issues such as the poor parallelism of network architecture, inability to flexibly capture long-term dependencies of features, and insufficient algorithm stability.

In this study, we developed a novel end-to-end circRNA-binding site prediction model called CircSSNN (CircRNA-binding site prediction via Sequence Self-attention Neural Network). To capture the hierarchical relationship between nucleotide sequences, we extract the initial features of circRNA sequence by a scheme of aggregating multiple gene encoding, including static local context and dynamic global context information. We then use the Transformer to design a network architecture i.e., Seq_Transformer, to extract the latent nucleotide dependencies to complete the task of CircRNA-RBP site prediction.

In the proposed model, the ResNet and LayerNorm modules are incorporated into the deep network to improve the robustness and reduce the sensitivity to hyperparameters, which also allows the algorithm to generalize well to different RNA-RBP combination recognition tasks. We compared CircSSNN with several state-of-the-art baselines on 37 popular circRNA benchmark datasets to verify its effectiveness and generalizability. Moreover, while keeping the network structure and hyperparameters unchanged, we directly applied CircSSNN to 31 linear RNAs datasets, and also obtained better performance than existing methods. The experimental results show that CircSSNN is superior to existing methods in terms of the recognition performance, and generalizability to different types of RNA-RBP. As such, it can serve as a competing candidate for the task of RNA-RBP prediction with a wide range of applications.

## Materials and methods

### Datasets

To verify the effectiveness of the CircSSNN, we adopted 37 circRNA datasets as benchmark datasets following the baselines we compared [15, 16]. We first downloaded the datasets from the circRNA interactome database (https://circinteractome.nia.nih.gov/). Subsequently, we obtained 335,976 positive samples and 335,976 negative samples following the process of iCircRBP-DHN [17].

To demonstrate the generalizability of CircSSNN regarding different types of RNA-RBP, we also tested the algorithm on 31 linear RNA datasets [24, 25] coming from CLIP-Seq data. Each linear RNA dataset has 5000 training samples and 1000 test samples [16].

### Feature multi-descriptors

In CircSSNN, all CircRNA fragments were encoded into three types of quantified features: KNFP for expressing different levels of local contextual features, CircRNA2Vec for capturing contextual features representing long-term dependencies, and DNABERT for describing the global embedding features with learnable position encoding.

### K-tuple nucleotide frequency pattern

To describe the local dependencies of circRNA sequences, KNFP is used to count the word frequency of substrings of circRNA with different lengths, thus the local context with varying lengths can be effectively captured [26].

**Fig. 1:**
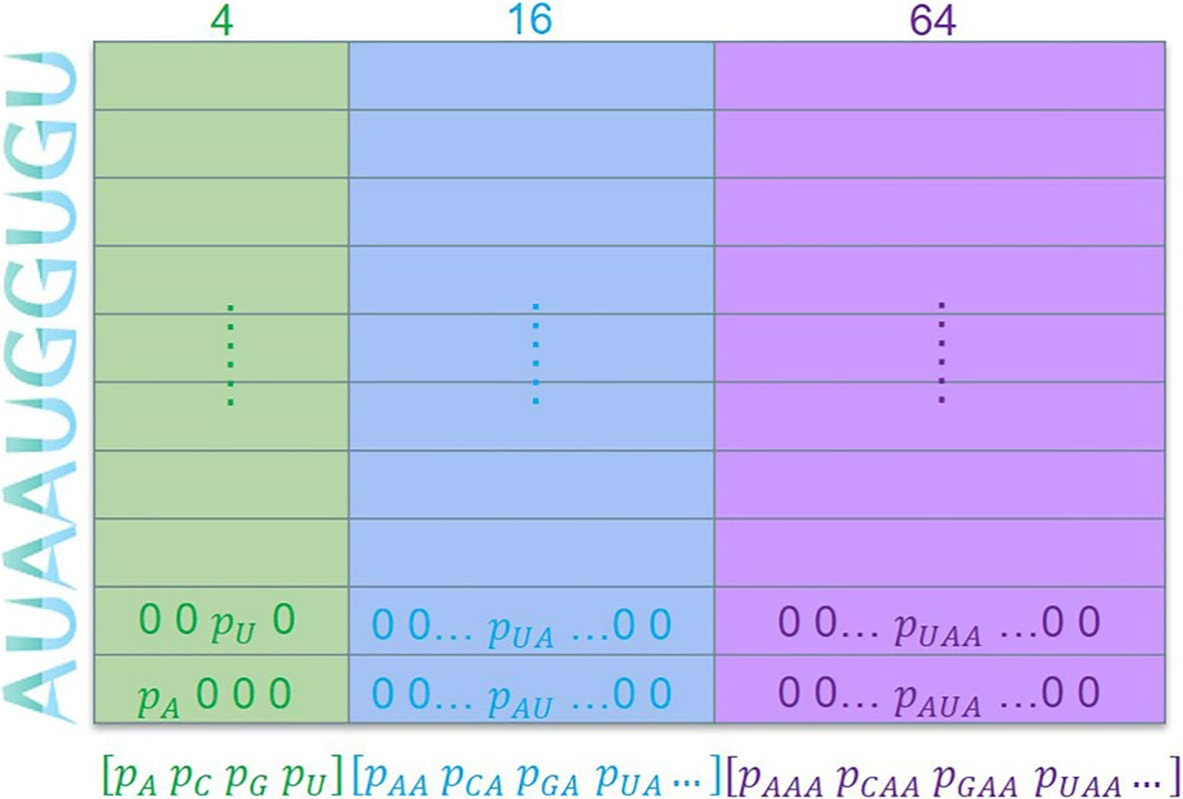
Encoding scheme of KNFP

Figure 1 shows the KNFP used in this paper consisting of three parts [17]: mononucleotide composition, dinucleotide composition and trinucleotide composition, i.e., k = 1,2,3. Considering a circRNA sequence with length *n*, i.e., $S = \left[ {S_{1} , S_{2} , \ldots S_{n} } \right]$, in which $S_{i} \in \left\{ {A,G,C,U} \right\}$, K-tuple nt composition can be employed to encode the raw sequence to get vector mixed by *P*_1_, *P*_2_, *P*_3_, in which each vector represents an individual k-tuple nt composition pattern, and it contains 4^* k*^ components as following:

1 $${\text{P}}_{k} = \left[ {p_{1} ,p_{2} ,p_{3} ,p_{4} , \ldots ,p_{{4^{k} }} } \right]$$

### CircRNA2Vec

We adopted the Doc2Vec model [27] to learn the global expression of circRNAs. Doc2Vec first obtains the circRNA substrings by moving a sliding window of width ten letter each step over the CircRNA sequence, and then tokenizes the obtained substrings into circRNA words by using the Circrna corpus from circBase [28].

We used Doc2Vec to learn the distributed expression of circRNA after tokenization. Specifically, for a central word *w*_*t*_ obtained by tokenization, considering its context words $w_{t - k} \sim w_{t + k}$, the conditional probability of this central word can be modeled as following,

2 $$\frac{1}{T}\sum\limits_{t = k}^{T - k} {\log } p\left( {w_{t} |w_{t - k} , \ldots ,w_{t + k} ,d} \right)$$

where d is the matrix of the document containing the substring considered, this is the difference between Doc2Vec and word2vec [29], i.e., the former considers the information of the document [27].

### Global embedding features based on CircRNA sequences

BERT is a language model that has achieved great success recently. Based on Transformer, BERT trains its network by using unsupervised learning. Different from word2vec and Doc2Vec, BERT contains learnable positional parameters and thus can express relative position in the context. Pre-training with BERT can obtain well-generalized base parameters, which can be applied to a specific task just with corresponding fine-tuning.

Similar to HCRNet [23], we first tokenized a circRNA sequence by k-mer in which k is set as 3. Next, we performed fine-tuning on a large amount of circRNA data. Similar to the original BERT, this pre-training and fine-tuning strategy will save a lot of training time and facilitate the following learning tasks remarkably.

### Deep neural network architecture

**Fig. 2:**
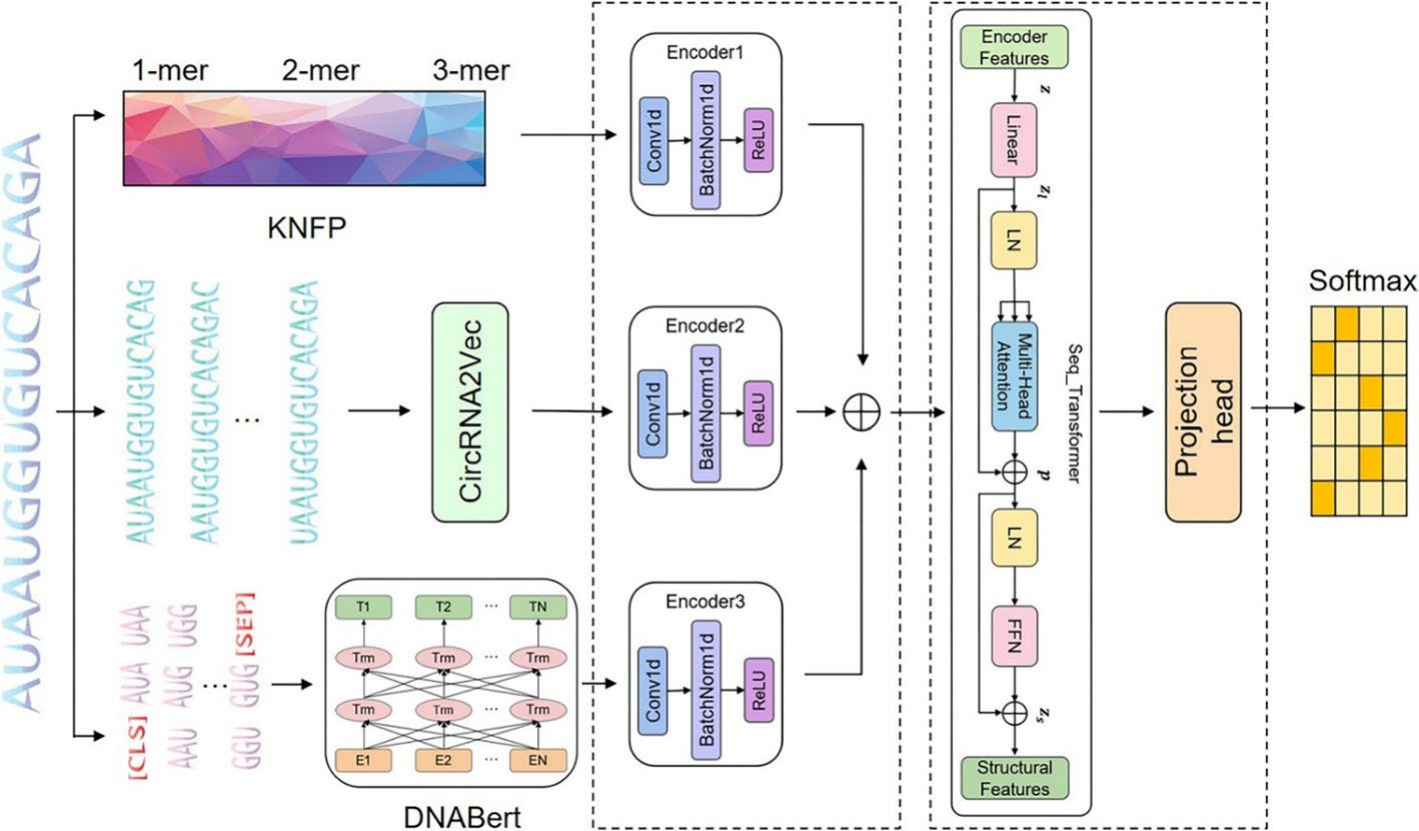
The network framework of the CircSSNN

**Fig. 3:**
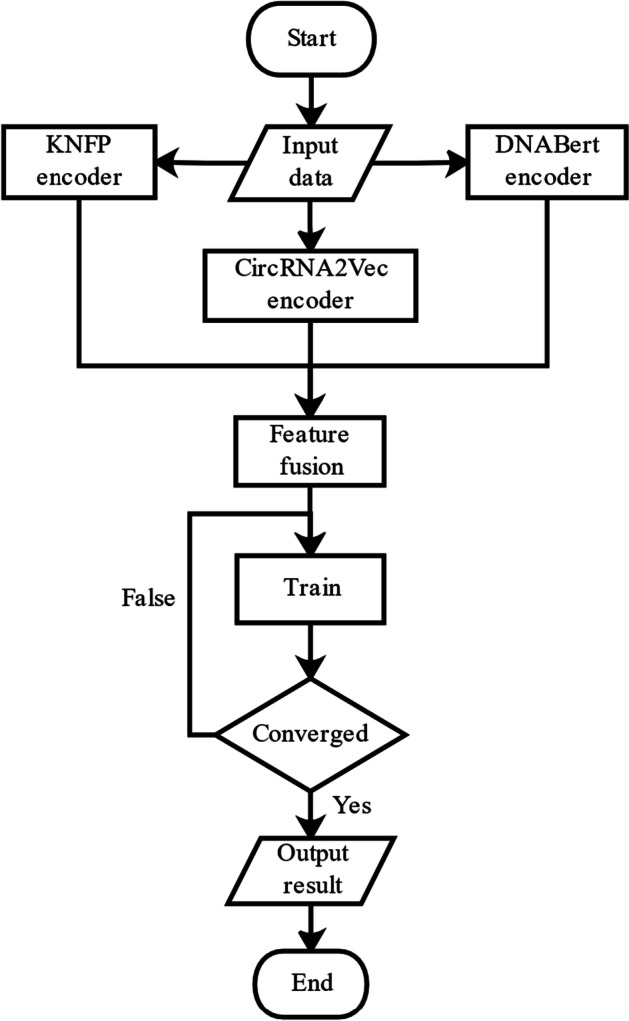
Experimental flowchart of the CircSSNN

In this section, we propose the CircSSNN framework to fully exploit the latent representation of features and facilitate the subsequent classification tasks. The overall framework of network is shown in Fig. 2. The CircSSNN consists of two parts in total, i.e., the feature encoding module and the Sequence Self-Attention Mechanism module. As stated above, multiple initial features are extracted from the raw sequence by KNFP, CircRNA2vec and DNABERT, and these initial features are first input into the feature encoding module to obtain the unified feature sequences, which are subsequently input into the next module to extract features with self-attention. The final step of classification is carried out by SoftMax. The experimental flowchart of the CircSSNN is illustrated in Fig. 3.

### Feature encoding module

The multiple initial features obtained from different feature descriptors have inconsistent channel numbers, magnitudes, magnitude units, etc. Such issues will hinder the later analysis. To overcome these issues, data unifying is needed to ensure that the initial features share the same form to facilitate the subsequent feature fusion.

We construct the feature encoder layer by CNN to unify the channels of multiple initial features and conduct data normalization. The feature encoder layer consists of three sublayers, i.e., the one-dimensional CNN layer, the one-dimensional BatchNorm layer, and the ReLU activation function.

### Sequence self-attention mechanism module

Transformer [30] is a network architecture based on attention mechanisms and abandoned traditional CNN and RNN. More precisely, a Transformer module consists only of Self-Attention and Feedforward Neural Network (FNN). This simple architecture of the Transformer brings better performance, higher parallelism, and less time-complexity. It has been successfully applied to various fields such as NLP and CV, and many researchers [31–33] have incorporated the Transformer as a sub-model and achieved impressive success.

**Fig. 4:**
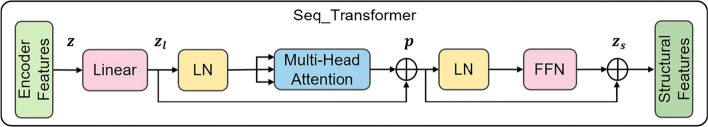
The structure of Seq_Transformer

We partially adopt the architecture of the Transformer with slight modification as the extractor of deep structure, i.e., the Seq_Transformer as shown in Fig. 4.

When constructing a neural network using the Transformer architecture superimposing multiple sub-layers, either in the encoder or in the decoder, leads to poor information propagation through the network, thus making the training very difficult [34, 35]. To overcome this issue, we leveraged the residual module to improve the efficiency of information propagation and conduct layer normalization to reduce the variance of the sub-layers. There are two ways to incorporate layer normalization into the residual network. Let *F* be a sub-layer (either in the encoder or decoder) in the Transformer architecture, and denote its parameter set by *θ*_*l*_.

#### Post-norm

In the pioneering works of the Transformer [30], it is common practice to do residual addition followed by Layer Normalization (LN) as follows,

3 $$y_{l} = x_{l} + {\text{F}}(x_{l} ;\theta_{l} )$$

4 $$x_{l + 1} = {\text{LN}}(y_{l} )$$

#### Pre-norm

In recent years, many researchers [36] prefer to conduct Layer Normalization (LN) on the inputs of sublayers rather than the outputs, like this,

5 $$x_{l + 1} = x_{l} + {\text{F}}({\text{LN}}(x_{l} );\theta_{l} )$$

The effect of Post-Norm or pre-Norm is comparable for shallow networks. Both methods can effectively improve the distribution of parameters, which facilitates smooth training. However, for a deeper network, it has been pointed out that Pre-norm is better than Post-norm [34, 35]. Specifically, for CircSSNN, since DNABERT is used in the initial feature extraction and the Seq_Transfomer is designed next, the network is rather deep in general. Therefore, for the cirRNA-RBP prediction, which is the task of the proposed model, we argue that the Pre-norm is more effective than the Post-norm. We have empirically demonstrated this point in the ablation experiments in the Section of Results.

Theoretically, this phenomenon can be explained by carefully examining of the nature of network training. It is well known that the training network is essentially the backward propagation of error computed by the loss function and the corresponding adjustment of weight parameters of the network according to the error propagation. Take a submodule containing L-layers for example, the error back-propagated from the next layer is represented by *ε*, and *x*_*L*_ represents the output of the last layer. If the Transformer adopts the Post-Norm strategy, according to the chain rule, the partial derivative of *ε* with respect to *x*_*L*_ can be calculated for a particular sublayer *x*_*l*_ as follows [35],

6 $$\frac{{\partial {\mathcal{E}}}}{{\partial x_{l} }} = \frac{{\partial {\mathcal{E}}}}{{\partial x_{L} }} \times \prod\limits_{k = l}^{L - 1} {\frac{{\partial {\mathbf{LN}}\left( {y_{k} } \right)}}{{\partial y_{k} }}} \times \prod\limits_{k = l}^{L - 1} {\left( {1 + \frac{{\partial {\text{F}}\left( {x_{k} ;\theta_{k} } \right)}}{{\partial x_{k} }}} \right)}$$

where $\prod\nolimits_{k = l}^{L - 1} {\frac{{\partial {\text{LN}}\left( {y_{k} } \right)}}{{\partial y_{k} }}}$ denotes the normalized information which is propagated backward, and $\prod\nolimits_{k = l}^{L - 1} {\left( {1 + \frac{{\partial {\text{F}}\left( {x_{k} ;\theta_{k} } \right)}}{{\partial x_{k} }}} \right)}$ indicates the information which is back-propagated through the residual module. Similarly, for the case of the Pre-norm, we can obtain the gradient as follows [35],

7 $$\frac{{\partial {\mathcal{E}}}}{{\partial x_{l} }} = \frac{{\partial {\mathcal{E}}}}{{\partial x_{L} }} \times \left( {1 + \sum\limits_{k = l}^{L - 1} {\frac{{\partial {\text{F}}\left( {{\text{LN}}\left( {x_{k} } \right);\theta_{k} } \right)}}{{\partial x_{l} }}} } \right)$$

From Eq. (7), it is easy to find out that the term “1” in the parenthesis enables the direct backward propagation of $\frac{{\partial {\mathcal{E}}}}{{\partial x_{L} }}$ from the last layer to the *l*th layer, i.e., the propagation through the residual module no longer depends on the number of layers.

Comparing the calculation of the information propagation of the residual module in Eq. (6) and Eq. (7), one can find that in Eq. (6) the information passing through the residual module does not propagate directly from layer L to layer *l*. This is because in Post-norm, the residual connection module is not a real bypass of the layer-normalization layer, resulting in a concatenated multiplicative term for the gradient propagation of the residual module in Eq. (6), i.e., $\prod\nolimits_{k = l}^{L - 1} {\frac{{\partial {\text{LN}}\left( {y_{k} } \right)}}{{\partial y_{k} }}}$, in which it can be found obviously, if the number of layers goes deeper, this term will suffer from gradient vanishing or exploding.

Therefore, our model is connected by Pre-norm residual blocks [34, 35], and features are normalized before passing through the multi-headed self-attention network, thus producing a more stable gradient.

The overall process of CircSSNN is as follows. We first extract multiple initial features using KNFP, CircRNA2vec, and DNABERT respectively. These initial features are then integrated into multi-view fused feature *z*_*l*_, which is divided into two ways using the residual connection module as follows,

8 $$p = z_{l} + {\text{MultiHeadAttention}}({\text{LN}} (z_{l} ))$$

In Eq. (8), one way of information remained as it was and propagated from right to left directly, while the other way of information was first normalized by Pre-norm LN before passing through the MHA module. The Pre-norm LN is defined as,

9 $$\mu = \frac{1}{M}\sum\limits_{i = 1}^{M} {z_{i} }$$

10 $$\sigma^{2} = \frac{1}{M}\sum\limits_{i = 1}^{M} {\left( {z_{i} - \mu } \right)^{2} }$$

11 $$\hat{z}=\frac{\mathbf{z}-\mu}{\sqrt{\sigma^2+\epsilon}} \odot \gamma+\beta \triangleq \operatorname{LN}_{\gamma_{,} \beta}(z)$$

In Eqs. (9–11), *M* is the number of neurons. Features are extracted using scaled dot-product multi-head attention to capture contextual features as follows,

12 $$Q = {\text{Concat}} \left( {q_{1} , \ldots ,q_{{\text{h}}} } \right)$$

13 $$K = {\text{Concat}} \left( {k_{1} , \ldots ,k_{{\text{h}}} } \right)$$

14 $$V = {\text{Concat}} \left( {v_{1} , \ldots ,v_{{\text{h}}} } \right)$$

15 $${\text{MultiHeadAttention}}(Q,K,V) = {\text{softmax}} \left( {\frac{{QK^{T} }}{\sqrt d }} \right)V$$

In Eqs. (12–14), *h* is the number of heads, *q*_*i*_, *k*_*i*_ and *v*_*i*_, $i \in \left\{ {1,2, \ldots h} \right\}$ denote the query, key, and value respectively. *Q*, *K* and *V* indicate the aggregation of multiple *q*_*i*_, *k*_*i*_*,* and *v*_*i*_, respectively. In Eq. (15), *d* is the dimension of the input vector. Then, the information passing through the MHA module and bypassing it are added together to get *p* as described in Eq. (8). Similarly, before the information passes through the FFN module, it is also processed by Pre-norm LN. In this way, the input information is finally turned into a unified structured deep feature to conduct the subsequent classification.

16 $$z_{s} = p + {\text{FFN}}({\text{LN}} (p))$$

From the network architecture of CircSSNN, one can find it differs from the existing models in two aspects.

First, to the best of our knowledge, this is the first attempt to introduce the residual module with Pre-norm LN in CircRNA recognition. As stated in [34, 35], the residual module with Post-norm LN brought about a higher risk of gradient vanishing or exploding when the network goes deeper. Therefore, we adopt the Pre-norm LN scheme to avoid this problem while using the residual connection to improve the efficiency of information transmission.

Second, we proposed the Seq_Transformer module based on self-attention to extract temporal contextual features. Most of the existing works proposed for CircRNA-RBP prediction, such as DMSK [20], CRBPDL [21], iCircRBP-DHN [17], and CRIP, etc., mainly use RNN such as LSTM or GRU for capturing temporal dependence. However, the computation of RNN or its variants is sequential, i.e., calculatiing results of time step *t* must depend on that of time step *t*-1, which dramatically limits the parallelism. In addition, long-term dependency is prone to loss during propagation along the sequential RNN network. LSTM and GRU adopted some gating mechanisms to mitigate this problem to a certain extent, but the effectiveness of gating mechanisms is undesirable for long-term dependencies. Therefore, compared with the models based on the Self-Attention mechanism, these models suffer from insufficient parallelism and poor ability to capture long dependencies. However, the attention mechanism has seldom been employed to extract features directly in this field. Up to now, only Yang et al. used the Attention mechanism in the iCircRBP-DHN model they proposed in 2020. But in iCircRBP-DHN, the attention mechanism was not employed as a direct feature extractor but as a supplement to the GRU mechanism, i.e., iCircRBP-DHN use the attention modules to capture features after GRU processing, which to some extent destroys the dependency relationship of the original data and makes the Attention mechanism play little role. In their subsequent work, i.e., the HCRNet proposed in 2022, they omitted the attention mechanism. In HCRNet, Yang et al. used DTCN to extract discriminative information from hybrid features and combine the parallelism of CNN with residual connection, and thus making various perceptual field sizes available and gradients stable. DTCN alleviates the limitations of RNN regarding to parallelism to some extent. However, it is still limited by the fixed perceptual field size of CNN, and the two issues of existing models, i.e., insufficient parallelism and inefficiency in capturing long-term dependencies, still exist. In contrast, in CircSSNN, after the initial multiple features were integrated into a unified one, feature extraction is performed directly using Seq_Transformer without intermediate processing by RNN or its variants. As a result, we solved the above two issues by adopting the Seq_Transformer. The advantages of Seq_Transformer can be analyzed as follows. First, it is constructed based on the Attention mechanism rather than sequential structure, so its calculation can be performed in the format of matrix multiplication, which can be easily parallelized and accelerated by modern deep learning frameworks based on GPUs. Second, by using the Seq_Transformer, the distance between any two positions in the sequence can be reduced to a constant, and long-term dependence can be effectively captured. In addition, due to the excellent parallelism of the Seq_Transformer, we can make the full use of multi-headed attention to focus on contextual information from different locations simultaneously. Therefore, the deep structure features extracted by the Seq_Transformer have good classification performance.

## Results

### Experimental setting

For both circRNA and linRNA datasets, 80% of the samples were randomly selected as training data. The remaining 20% of them were used as test data. To show the generalizability of CircSSNN rather than the performance improvement brought by hyperparameter tuning, we didn’t set validation sets for hyperparameter tuning in experiments. The hyperparameters of CircSSNN were set to be the same across all datasets, which eliminates the trouble of hyperparameters tuning.

We used Adam as the optimizer, and set the parameters weight_decay and batch_size as 3e-4 and 64 respectively. The learning rate of Adam was controlled by the built-in learning rate scheduler of Pytorch in which the parameter *initial_rate* was set to be 3e-3. As the Seq_Transformer can capture deep features effectively and quickly, we let the learning rate decay to one-tenth every two rounds to accelerate the convergence.

### Experimental results on circRNA datasets

We compared the CircSSNN with seven baselines on 37 circRNA-RBP datasets. To be fair, all the parameters were set as reported in the corresponding papers.

**Fig. 5:**
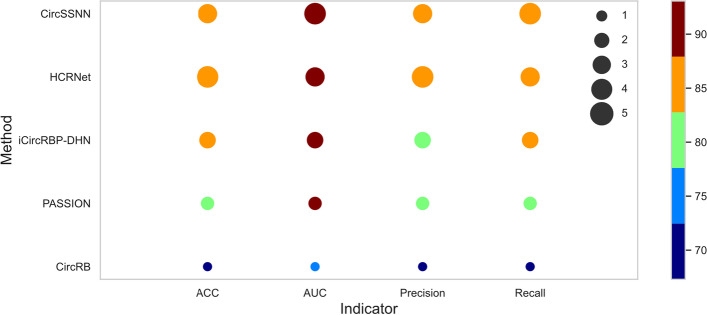
The average performance of competing methods on 37 circRNA datasets

Four metrics including AUC, ACC, precision, and recall, were used to compare the performance of the competing methods. The performances of all methods, averaging over 37 circRNA datasets, were shown in Fig. 5. In Fig. 5, the colors of the solid circles correspond to the performance of each algorithm with respect to a certain metric, and these numbers can be obtained by looking at the color bars on the right side of Fig. 5, e.g., the green solid circle in the fourth row of the last column (from top to bottom) represents the performance of the PASSION model with respect to recall, which is about 80% (the third block in the color bar). The size of the circles indicates the ranking of the performances, i.e., the largest circle of size 5 corresponds to the best algorithm for each metric, while the smallest circle of size 1 corresponds to the worst one. Take the last column as an example again, since the Recall of CircSSNN, HCRNet and iCircRBP all are around 85% (the same color), but the size of the solid circles gives their ranking, i.e., in terms of recall, CircSSNN has the best performance among the three algorithms and iCircRBP has the worst performance.

As can be seen from Fig. 5, the performance of CircSSNN is superior to all competing methods regarding to AUC and Recall, and is slightly inferior to HCRNet regarding ACC and Precision, but is higher than the other six methods by a large margin. The detailed average value of different methods regarding ACC, AUC, Precision and recall are 85.71%, 93.07%, 85.14%, 86.69% for CircSSNN; 85.81%, 93.04%, 85.68%, 86.35% for HCRNet. As the performances of other baselines are obviously by far inferior to that of the two methods mentioned above, we don’t list them here for simplicity. The detailed AUC values are summarized in Table 1.

**Table 1:** The AUC of competing methods on 37 circRNA datasets

Dataset	CircSSNN	HCRNet	CRBPDL	iCircRBP-DHN	DeCban	PASSION	CircRB	circ2CBA
AGO1	0.92 ± 0.004	0.929 ± 0.017	0.9232	0.898 ± 0.003	0.873	0.909 ± 0.003	0.750 ± 0.003	0.9009
AGO2	0.847 ± 0.037	0.856 ± 0.236	0.8233	0.797 ± 0.004	0.846	0.822 ± 0.003	0.624 ± 0.008	0.8029
AGO3	0.949 ± 0.006	0.941 ± 0.034	0.9472	0.920 ± 0.016	0.89	0.909 ± 0.008	0.718 ± 0.004	0.9105
ALKBH5	0.99 ± 0.033	0.989 ± 0.013	0.9952	0.979 ± 0.004	0.919	0.752 ± 0.030	0.593 ± 0.009	0.7696
AUF1	0.987 ± 0.003	0.988 ± 0.013	0.981	0.985 ± 0.002	0.959	0.979 ± 0.003	0.938 ± 0.003	0.9888
C17ORF85	0.982 ± 0.05	0.981 ± 0.047	0.9881	0.987 ± 0.002	0.915	0.860 ± 0.021	0.634 ± 0.021	–
C22ORF28	0.947 ± 0.073	0.936 ± 0.101	0.9088	0.913 ± 0.004	0.877	0.894 ± 0.008	0.731 ± 0.005	–
CAPRIN1	0.912 ± 0.007	0.904 ± 0.006	0.8765	0.858 ± 0.012	0.887	0.860 ± 0.009	0.685 ± 0.002	–
DGCR8	0.915 ± 0.054	0.917 ± 0.305	0.9236	0.906 ± 0.002	0.847	0.917 ± 0.002	0.770 ± 0.002	–
EIF4A3	0.847 ± 0.03	0.848 ± 0.035	0.853	0.799 ± 0.003	0.819	0.823 ± 0.004	0.662 ± 0.002	–
EWSR1	0.955 ± 0.011	0.949 ± 0.086	0.9443	0.942 ± 0.004	0.938	0.938 ± 0.006	0.805 ± 0.004	–
FMRP	0.925 ± 0.027	0.933 ± 0.02	0.8966	0.892 ± 0.002	0.845	0.900 ± 0.002	0.737 ± 0.001	–
FOX2	0.962 ± 0.005	0.961 ± 0.136	0.9618	0.958 ± 0.005	0.936	0.830 ± 0.034	0.535 ± 0.003	–
FUS	0.888 ± 0.002	0.888 ± 0.008	0.8618	0.855 ± 0.004	0.842	0.859 ± 0.002	0.697 ± 0.004	–
FXR1	0.995 ± 0.013	0.984 ± 0.019	0.9948	0.994 ± 0.001	0.934	0.959 ± 0.009	0.838 ± 0.015	0.9579
FXR2	0.966 ± 0.043	0.96 ± 0.023	0.9518	0.939 ± 0.009	0.909	0.941 ± 0.003	0.774 ± 0.003	–
HNRNPC	0.973 ± 0.002	0.981 ± 0.002	0.9771	0.977 ± 0.001	0.97	0.976 ± 0.001	0.941 ± 0.003	–
HUR	0.908 ± 0.011	0.906 ± 0.009	0.8758	0.867 ± 0.005	0.841	0.879 ± 0.006	0.666 ± 0.001	0.8741
IGF2BP1	0.889 ± 0.019	0.886 ± 0.021	0.8554	0.843 ± 0.002	0.859	0.845 ± 0.003	0.679 ± 0.003	–
IGF2BP2	0.871 ± 0.004	0.875 ± 0.082	0.8426	0.831 ± 0.004	0.886	0.827 ± 0.009	0.644 ± 0.009	–
IGF2BP3	0.867 ± 0.006	0.871 ± 0.298	0.8229	0.816 ± 0.004	0.867	0.831 ± 0.003	0.635 ± 0.004	–
LIN28A	0.897 ± 0.014	0.901 ± 0.071	0.8751	0.857 ± 0.007	0.871	0.875 ± 0.005	0.671 ± 0.003	–
LIN28B	0.919 ± 0.038	0.912 ± 0.178	0.9014	0.892 ± 0.004	0.893	0.889 ± 0.005	0.731 ± 0.003	–
METTL3	0.892 ± 0.147	0.901 ± 0.365	0.8649	0.852 ± 0.009	0.918	0.878 ± 0.010	0.731 ± 0.006	–
MOV10	0.87 ± 0.207	0.866 ± 0.498	0.8674	0.838 ± 0.006	0.958	0.845 ± 0.005	0.698 ± 0.004	–
PTB	0.854 ± 0.042	0.851 ± 0.096	0.8347	0.822 ± 0.006	0.873	0.829 ± 0.004	0.663 ± 0.002	–
PUM2	0.974 ± 0.053	0.979 ± 0.041	0.9758	0.970 ± 0.004	0.959	0.952 ± 0.004	0.854 ± 0.001	–
QKI	0.988 ± 0.005	0.989 ± 0.013	0.9879	0.971 ± 0.006	0.933	0.927 ± 0.005	0.807 ± 0.006	–
SFRS1	0.981 ± 0.001	0.979 ± 0.002	0.9684	0.964 ± 0.000	0.891	0.965 ± 0.003	0.836 ± 0.003	–
TAF15	0.996 ± 0.001	0.996 ± 0.003	0.9945	0.992 ± 0.002	0.925	0.967 ± 0.002	0.883 ± 0.006	0.9851
TDP43	0.932 ± 0.043	0.939 ± 0.058	0.9336	0.926 ± 0.002	0.964	0.934 ± 0.002	0.829 ± 0.004	–
TIA1	0.982 ± 0.091	0.972 ± 0.009	0.9666	0.961 ± 0.004	0.967	0.935 ± 0.006	0.827 ± 0.008	–
TIAL1	0.936 ± 0.067	0.921 ± 0.241	0.9249	0.917 ± 0.003	0.965	0.906 ± 0.003	0.820 ± 0.004	–
TNRC6	0.952 ± 0.016	0.953 ± 0.124	0.9797	0.967 ± 0.002	0.925	0.785 ± 0.010	0.550 ± 0.017	–
U2AF65	0.934 ± 0.036	0.932 ± 0.104	0.9306	0.926 ± 0.002	0.931	0.930 ± 0.002	0.787 ± 0.003	–
WTAP	0.973 ± 0.007	0.983 ± 0.86	0.9713	0.967 ± 0.006	0.934	0.794 ± 0.069	0.621 ± 0.025	–
ZC3H7B	0.855 ± 0.06	0.863 ± 0.021	0.8151	0.804 ± 0.003	0.909	0.804 ± 0.005	0.634 ± 0.006	–
AVG	0.931 ± 0.054	0.93 ± 0.071	0.9188	0.908 ± 0.060	0.9047	0.884 ± 0.060	0.729 ± 0.102	–

Apparently, CircSSNN outperformed other competing baselines on 18 out of 37 circRNAs datasets, and produced the highest average AUC of 93.1%. The number of samples in each the 37 benchmark datasets ranges from 892 to 40,000, which validates that CircSSNN is applicable for datasets with an extensive range of scales. Even for small-scale datasets, CircSSNN still achieved competing performance.

**Fig. 6:**
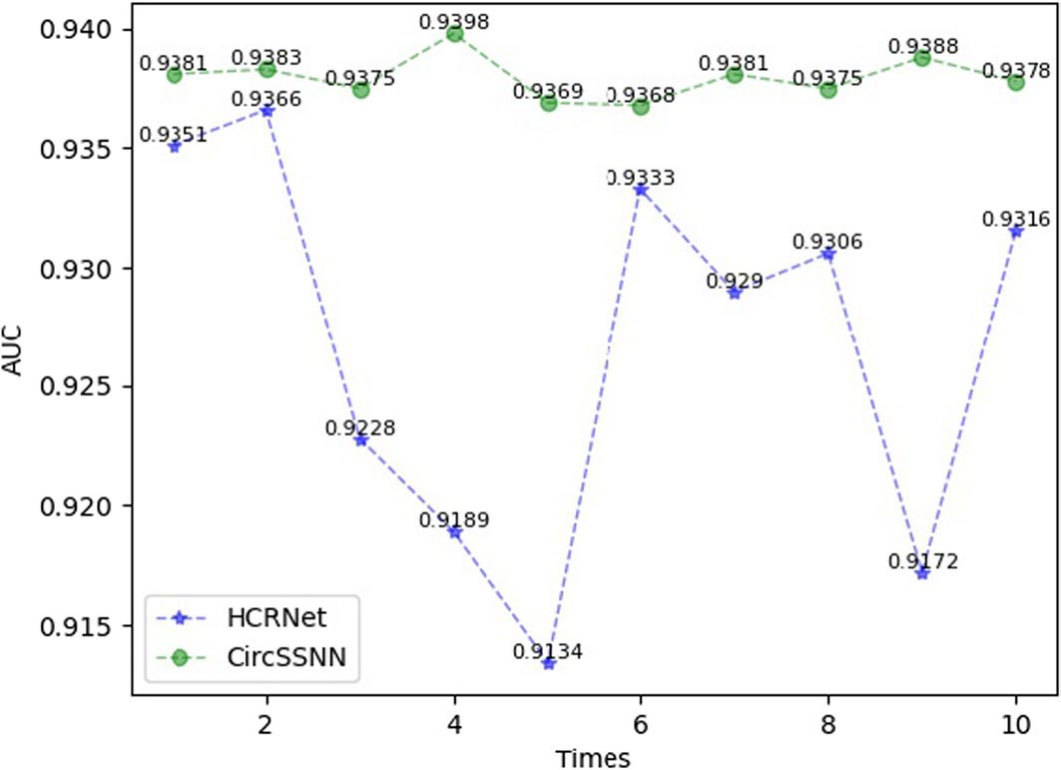
Comparison of the stability of HCRNet

To demonstrate the stability of the CircSSNN, we selected a moderate-scale dataset TIAL1 with 10,912 samples, and repeated the test of the top two models, i.e., CircSSNN and HCRNet, ten times on TIAL1. The fluctuation of performance was illustrated in Fig. 6. In Fig. 6, the curve of CircSSNN fluctuated more mildly than that of the HCRNet. It further illustrated that the Seq_Transformer used in the CircSSNN was more flexible, and less affected by sample randomness, and the features extracted by the Seq_Transformer are more stable.

To compare the efficiency and parallelism of the CircSSNN and HCRNet, we trained the two models on 37 circRNA datasets ten times with the same hardware and software configuration, and the results showed the average training times of the two models are 10 h and 13 h, respectively, which showed that CircSSNN was more efficient and parallelizable. The reason is that the Seq_transformer used in the CircSSNN is entirely based on the attention mechanism, which converts data into Query, Key, and Value at the same time, and thus facilitates the parallel retrieval of feature information.

**Fig. 7:**
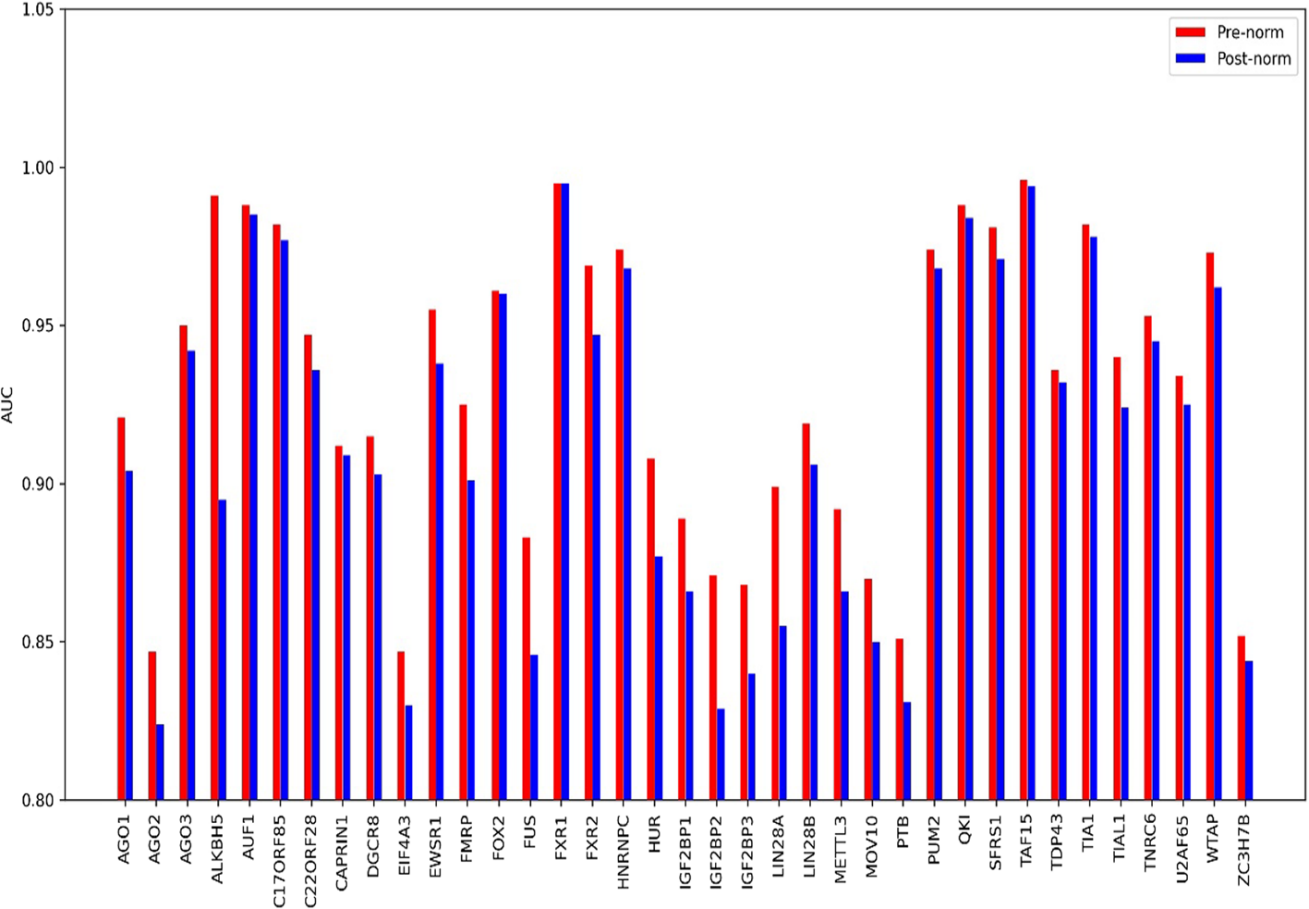
Comparison of the effect of pre-norm and post-norm on 37 circRNA datasets

To demonstrate the advantage of Pre-norm over Post-norm, we kept the other modules of the CircSSNN unchanged, and compared the effect of Pre-norm and Post-norm on 37 circRNA datasets. In Fig. 7, the blue bar represents the performance of the CircSSNN with the Post-norm strategy, while the red bar represents the performance of the Pre-norm. As shown in Fig. 7, the Pre-norm strategy brings performance gains on 36 out of 37 datasets, with an increase of more than two percents on about half of the datasets.

**Fig. 8:**
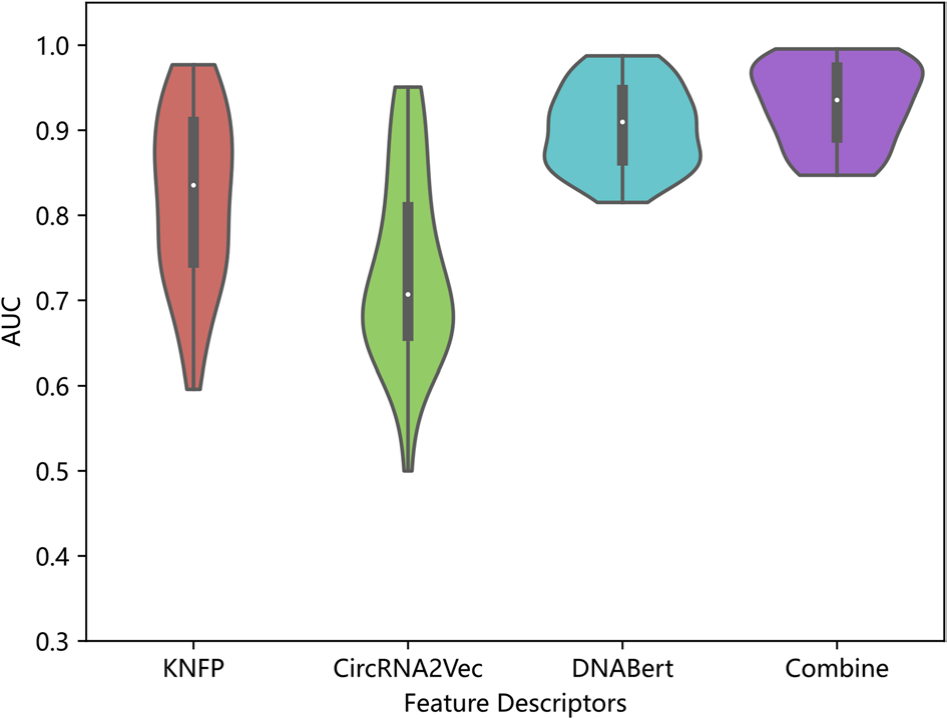
Comparison of the effect of different feature descriptors

Finally, to demonstrate that the proposed feature fusion scheme is more effective than a single feature descriptor, ablation experiments were conducted while keeping other modules (except the feature descriptors modules) unchanged, and the results were plotted as violin plots, as shown in Fig. 8. It can be seen that, in terms of the AUC values of the proposed algorithm on 37 circRNA datasets, the distribution of the results obtained by the feature fusion scheme is more concentrated compared to that of a single feature descriptor, and the mean AUC values obtained by the feature fusion scheme are also the largest. The performance of the two descriptors, KNFP and CircRNA2Vec, varies obviously across different datasets, while the results of DNABert descriptors are more evenly distributed compared to the previous two, but its performance is also slightly inferior compared to the results of the feature fusion scheme. From Fig. 8, it can be seen that feature fusion scheme makes full use of the consistent and complementary information of each view and obtains excellent overall performance.

### The prediction performance of CircSSNN on linear RNA datasets

**Fig. 9:**
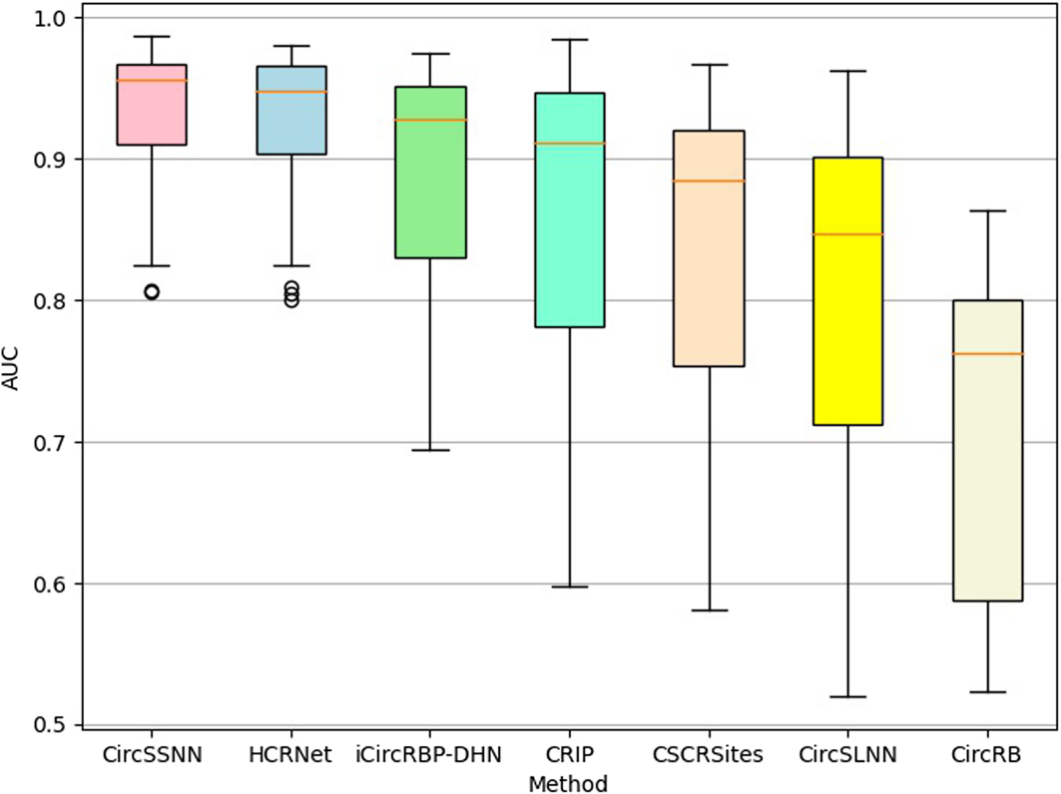
Boxplot comparison results of different models on 31 linear RNA datasets regarding to AUC

The CircSSNN is highly transformable, and can be applied to other types of RNA-RBP prediction tasks without hyperparameters tuning. To verify this, we tested the CircSSNN and the baselines on 31 linear RNA datasets, and the results were shown in Fig. 9. As shown in Fig. 9, without hyperparameters tuning, the CircSSNN achieved favorable performance over other state-of-the-art baselines, which demonstrated the CircSSNN was stable and transformable. The detailed value of AUC was listed in Table 2. Because the models designed for the cirRNA datasets, such as HCRNet and iCircRBP-DHN, do not specify the necessary details of operation and parameter settings for migrating them from the cirRNA dataset to the linRNA dataset, we cannot reproduce the results of these models in our experiments, and just list in Table 2 the AUC values published in their original papers for comparison. However, as can be observed in Fig. 9 and Table 2, even though compared with their results which were produced after fine-tuning of hyperparameters with validate sets, the results of the CircSSNN, which was obtained without hyperparameters tuning, still outperformed these models in most cases. In detail, the proposed CircSSNN achieved the best AUC on 21 out of the 31 linear RNA datasets, and the average value of AUC is 0.931, which is 0.7 percent higher than that of the HCRNet. In some datasets, the CircSSNN outperforms the HCRNet quite a bit, for example, the AUC of the CircSSNN is 4.5 and 3.6 percent higher than the HCRNet on the hnRNPL 1 dataset and the hnRNPL-2 dataset, respectively. Therefore, even directly keeping unchanged the network architecture and parameters designed for circRNA datasets, the CircSSNN can still produce competitive results when applied to linear RNA datasets.

**Table 2:** Average value of AUC obtained by different methods on 31 linear RNA datasets

Dataset	CircSSNN	HCRNet	iCircRBP-DHN	CRIP	CSCRSites	CircSLNN	CircRB
AGO1234	**0.907**	**0.909**	0.788	0.737	0.708	0.662	0.588
AGO2-M	**0.825**	**0.809**	0.736	0.598	0.583	0.557	0.538
Binding_1	**0.968**	**0.967**	0.925	0.862	0.842	0.795	0.588
Binding_2	**0.972**	**0.959**	0.929	0.852	0.828	0.754	0.676
AGO2	**0.805**	**0.804**	0.800	0.638	0.636	0.562	0.609
eIF4AIII_1	**0.975**	**0.970**	0.963	0.952	0.937	0.894	0.769
eIF4AIII_2	**0.979**	**0.973**	0.963	0.954	0.944	0.897	0.775
ELVAL1-1	**0.956**	**0.946**	0.939	0.918	0.910	0.882	0.808
ELVAL1-M	**0.807**	**0.825**	0.695	0.604	0.581	0.520	0.525
ELVAL1-A	**0.937**	**0.938**	0.922	0.898	0.876	0.845	0.762
ELVAL1-2	**0.966**	**0.954**	0.943	0.926	0.925	0.898	0.784
EWSR1	**0.943**	**0.937**	0.918	0.912	0.884	0.851	0.765
FUS	**0.949**	**0.951**	0.947	0.941	0.907	0.905	0.791
mut-FUS	**0.960**	**0.961**	0.946	0.939	0.907	0.907	0.760
IGF2BP1-3	**0.891**	**0.888**	0.781	0.693	0.703	0.597	0.523
hnRNPC-1	**0.973**	**0.965**	0.952	0.963	0.936	0.935	0.862
hnRNPC-2	**0.987**	0.980	0.974	**0.985**	0.967	0.962	0.863
hnRNPL-1	**0.891**	**0.842**	0.829	0.748	0.650	0.670	0.584
hnRNPL-2	**0.836**	**0.800**	0.761	0.740	0.636	0.654	0.583
HnRNPL-L	**0.835**	**0.824**	0.779	0.685	0.632	0.636	0.555
MOV10	**0.932**	**0.919**	0.885	0.814	0.803	0.764	0.588
NSUN2	**0.913**	**0.898**	0.832	0.865	0.798	0.776	0.672
PUM2	**0.969**	**0.977**	0.969	0.963	0.959	0.920	0.814
QKI	0.963	**0.971**	0.962	**0.967**	0.956	0.929	0.818
SFRS1	**0.939**	**0.941**	0.912	0.886	0.885	0.794	0.659
TAF1S	0.966	**0.972**	**0.971**	0.963	0.922	0.925	0.796
TDP-43	**0.961**	**0.948**	0.928	0.911	0.913	0.841	0.762
TIA1	**0.966**	**0.955**	0.945	0.930	0.891	0.894	0.817
TIAL1	**0.941**	**0.928**	0.915	0.898	0.864	0.847	0.804
U2AF65	**0.981**	**0.978**	0.971	0.968	0.918	0.932	0.852
Y2AF65	**0.961**	**0.962**	0.951	0.935	0.906	0.893	0.792
Avg	**0.931**	**0.924**	0.895	0.860	0.832	0.803	0.712

The top-2 results of every column are highlighted in bold

**Fig. 10:**
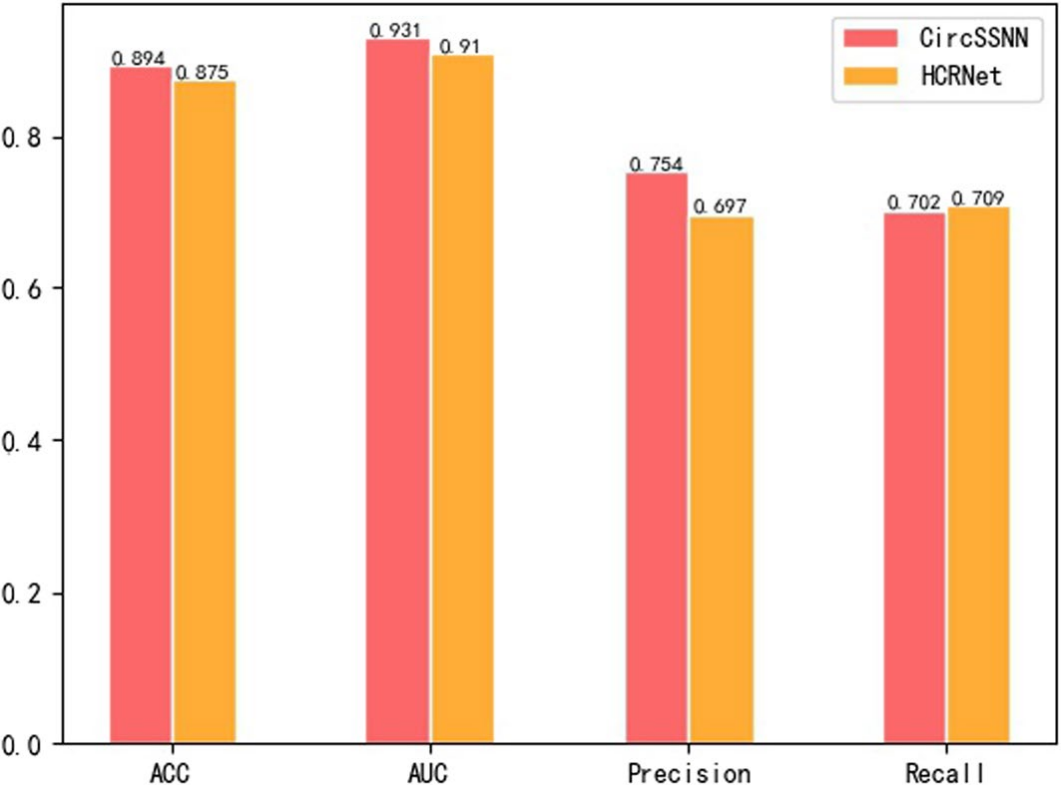
Comparison of CircSSNN and HCRNet on 31 linear RNA datasets

In addition, to investigate the transformability of different methods, we also compared the CircSSNN and the HCRNet, the newest and most representative algorithm, on linear RNA with their hyper-parameters setting on CircRNA. The experimental results on the 31 linear RNA benchmark datasets are shown in Fig. 10.

As shown in Fig. 10, when both the CircSSNN and the HCRNet were tested on the linRNA datasets with their hyper-parameters settings on the CircRNA datasets, the CircSSNN outperformed the HCRNet about two, two and six percent regarding ACC, AUC, and Precision, respectively, while just slightly inferior to HCRNet regarding Recall by 0.7 percent. These results verified that the CircSSNN was more transformable than the HCRNet, and was able to obtain favorable results even without hyperparameter tuning. The AUC of the HCRNet was reported as 0.924 in its original paper, which was the result obtained by fine-tuning the hyperparameters with validate sets, but it dropped to 0.91 when no task-oriented fine-tuning of hype-parameters was conducted. Therefore, although HCRNet also achieved good performance on the linRNA datasets, the tuning of its hyperparameters requires expertise and a lot of trial and error, which is not conducive to generalization. In contrast, CircSSNN can be simply and efficiently transformed to other RNA-RBP identification tasks and has a wide range of applications.

## Discussion

The above experimental results verify the Seq_transformer adopted in the CircSSNN can effectively capture the semantic and global context of sequences and produce discriminative features, and the CircSSNN is more parallelable, stable and transformable than other baselines.

Compared with existing methods, the CircSSNN network architecture proposed in this paper can achieve excellent performance for the following two reasons: First, after integrating data from multiple views, directly use Seq_Transformer and make full use of multiple attention to simultaneously pay attention to contextual information from different locations to extract deep features. Without intermediate processing by RNNs or their variants. The distance between any two positions in the sequence can be reduced to a constant, effectively capturing long-term dependencies. Second, the Pre-norm based attention mechanism first applied to CircRNA recognition task can avoid the gradient disappearance or explosion risk brought by deep network, so that network training can obtain more stable gradient update.

Although the improvement of the CircSSNN over the HCRNet was not very remarkable, the HCRNet needed to tune its hyperparameters by validation sets, which is time-consuming and laborious. In contrast, the CircSSNN used the same set of hyperparameters for all datasets, i.e., it didn’t need validation sets to fine-tune the hyperparameters, which demonstrated that the CircSSNN was more flexible and insensitive to hyperparameters. This appealing characteristic made it easier to use, especially for non-computer professionals.

## Conclusion

At present, most existing models for circRNA-RBP identification adopt CNN, RNN or their variant as feature extractors and have drawbacks such as poor parallelism, insufficient stability, and inability to capture long-term dependence. We propose the CircSSNN model based on the sequence self-attention mechanism. The CircSSNN extract deep features completely by the self-attention mechanism with good parallelism and can capture the long-term dependencies by reducing the distance between any two positions in a sequence to a constant. Multiple experiments on 37 circRNAs datasets and 31 linRNAs datasets using the same hyperparameters show that the CircSSNN achieves excellent performance, has good stability and scalability, and eliminates the problem of hyperparameters tuning compared with existing models. In conclusion, CircSSNN can serve as an appealing option for the task of circRNA-RBP identification.

## Data Availability

The datasets and codes are available at https://github.com/cc646201081/CircSSNN.

## Abbreviations

circRNAs: Circular RNAs
RBPs: RNA-binding proteins
CNN: Convolution neural network
RNN: Recurrent neural network
miRNA: MicroRNA
DTCN: Deep temporal convolutional network
KNFP: K-tuple Nucleotide Frequency Pattern
BERT: Bidirectional Encoder Representations from Transformers
CircSSNN: CircRNA-binding site prediction via Sequence Self-attention Neural Network
